# Manual Testicular Detorsion under Propofol Sedation

**DOI:** 10.1155/2009/529346

**Published:** 2009-10-08

**Authors:** Martyn Harvey, Giles Chanwai, Grant Cave

**Affiliations:** ^1^Department of Emergency Medicine, Waikato Hospital, Pembroke St, Hamilton 3240, New Zealand; ^2^Hutt Hospital, High Street, Lower Hutt 5010, New Zealand

## Abstract

Acute spermatic cord torsion is a urologic emergency requiring accurate diagnosis and timely intervention to effect testicular salvage. We report a case of adolescent testicular torsion successfully reduced following manual detorsion under sedation at the Emergency Department. Scrotal exploration and bilateral orchidopexy were subsequently performed as a semielective procedure.

## 1. Case Report

A 14-year-old adolescent male presented to our Emergency Department with sudden onset severe right testicular pain and associated nausea of one-hour's duration. Further history elicited no history of recent trauma and no suspicion of sexually transmitted infection. Physical examination was significant for pallor, bradycardia (pulse 59 bpm), and a tender right hemiscrotum. The testis on the right was exquisitely tender and noted to be both high-riding and displayed a transverse longitudinal-axis lie. Testicluar pain was exacerbated by scrotal elevation, and the cremasteric reflex was absent. There was no scrotal oedema and no urethral discharge. The remainder of the physical examination proved noncontributory. Bedside urinanalysis was unremarkable. Given the typical presentation and clinical findings, testicular ultrasonography was not undertaken. Acute right-sided spermatic cord torsion (SCT) was diagnosed on clinical findings alone.

The patient subsequently underwent procedural sedation with fentanyl at 50 mcg and propofol titrated to 90 mg and manual detorsion of the right testis. Rotation of the testicle 180 degrees in an anticlockwise direction resulted in return of normal scrotal anatomy with vertical long axis orientation of the testis. On waking the patient reported complete resolution of testicular pain.

Following urologic consultation the patient was allowed home and returned the following day for elective scrotal exploration and bilateral orchidopexy. Operative findings revealed a healthy appearing right testicle and bilateral Bell-Clapper deformity. Both testicles were fixed at upper, middle, and lower poles. Postoperative recovery was uneventful, and physical examination at one-month follow-up revealed normal scrotal and testicular anatomic appearance.

## 2. Discussion

Spermatic cord torsion represents an acute urologic emergency. Testicular survival without atrophy is a function of the length of organ ischaemia. Testicular viability has been shown both experimentally [1] and clinically [2, 3] to correlate directly with duration of ischaemia. Most testes may be salvaged that if detorsion is effected within 6 hours of onset [3], conversely progressively more testicles are lost as the period of ischaemia lengthens. Testicular salvage is unusual after 10 to 12 hours of SCT and approaches zero at 24 hours [4]. There is however no absolute time limit beyond which complete testicular infarction may be assumed, since the degree of vascular occlusion may be variable.

Torsion of the spermatic cord usually results from initial lateral to medial rotation of the testicle. However, considerable variation has been reported. Medial rotation was reported in 108 of 162 (67%) and 20 of 28 (71%) cases of torsion by Sessions et al. [5] and Ransler III and Allen [6], respectively. Harrison [7] further report medial rotation in 7 of 8 patients with acute SCT. In addition to rotation in the horizontal plane, an element of caudal to cranial rotation with resulting cremasteric muscle spasm frequently serves to lock the testicle in position after rotation of greater than 180 degrees [4].

Most patients with confirmed or suspected SCT undergo emergent scrotal exploration with detorsion under direct vision followed by bilateral orchidopexy when vascularity is confirmed, or orchidectomy and contralateral orchidopexy if testicular infarction is present. The key to testicular preservation is however not an emergency operation per se, but detorsion of the spermatic cord and restoration of organ perfusion. This may be effected either preoperatively by manual reduction or intraoperatively under direct visualization.

Manipulative reduction of SCT was first described by Nash in 1893 [8]. Van der Poel later reported a 25-year-old physician suffering recurrent testicular torsion who would himself effect reduction manually [9]. Since these early reports, preoperative manual detorsion of the spermatic cord has been sporadically included in management algorithms for SCT with variable emphasis of the maneuver.

Manual detorsion requires reversal of the initial twisting process via rotation of the torsed testicle through 2 planes. Given the preponderance of medial rotation initial attempts at reduction should involve rotation of the testicle in a caudal to cranial direction to release the locking mechanism, in addition to simultaneous medial to lateral rotation to effect detorsion of the spermatic cord. The number of rotations required to successfully achieve reduction is likewise variable with up to 1080 horizontal degrees (three full turns) reportedly necessary in some cases. 

Recommendations for reduction advocate sequential rotation of the testicle by 180 degrees in one direction (initially lateral in accordance with given preponderance of medial direction of torsion), then maintaining the twist and attempting to turn the testis a further 180 degrees or more as deemed necessary. Increased pain or increased resistance to rotation is indications for attempting detorsion in the opposite direction. Successful reduction is reliably confirmed by lengthening of the spermatic cord, resolution of epididymal and spermatic cord oedema, a return to the anatomical position of the testicle, and near complete relief of testicular pain [4].

Successful preoperative manual detorsion has been reported in a number of case series. Cornel and Karthaus report successful reduction in 14 of 17 patients with acute unilateral SCT [10]. Similarly Kiesling et al. [4] and Cattolica et al. [2] further report successful manual detorsion and subsequent testicular survival in 94% (15 of 16) and 100% (34 of 34) of evaluable cases, respectively. Doppler ultrasound has been furthermore utlilised as an adjunct to guide reduction of SCT. Betts et al. report successful detorsion in eight of eleven cases of acute testicular torsion where USS was utilised to confirm restoration of blood flow on completion of the maneuver [11]. The performance of a doppler study however inherently necessitates both an experienced examiner and ready availability of compatible machine.

Success at manual detorsion has been demonstrated to decline with increasing duration of torsion. Greatest efficacy has been reported at initial physical examination. With passage of time however progressive scrotal oedema, induration, and adhesions from epididymitis or testicular infarction may serve to obscure vital landmarks and render manual detorsion difficult. Manual reduction may furthermore be augmented with either sedation as reported in the present case or regional anaesthesiae with spermatic cord blockade [4]. If detorsion is successfully achieved, subsequent scrotal exploration and orchidopexy may be considered an urgent or semielective procedure as opposed emergent in the event of manipulative failure. 

Spermatic cord torsion represents a commonly encountered surgical emergency wherein accurate diagnosis and timely intervention are essential to effecting testicular salvage. Whilst ideal treatment is immediate surgical exploration and detorsion under direct vision, Clinicians should be cognizant of manual detorting maneuvers and be prepared to institute these when surgical options are delayed or unavailable.
